# Effects of pen enrichment on leg health of fast and slower-growing broiler chickens

**DOI:** 10.1371/journal.pone.0254462

**Published:** 2021-12-23

**Authors:** Bahadır Can Güz, Ingrid C. de Jong, Carol Souza Da Silva, Fleur Veldkamp, Bas Kemp, Roos Molenaar, Henry van den Brand

**Affiliations:** 1 Adaptation Physiology Group, Wageningen University and Research, Wageningen, Gelderland, The Netherlands; 2 Wageningen Livestock Research, Wageningen University and Research, Wageningen, Gelderland, The Netherlands; Tokat Gaziosmanpasa Universitesi, TURKEY

## Abstract

Pen enrichment for broiler chickens is one of the potential strategies to stimulate locomotion and consequently contribute to better leg health and welfare. This study was designed to evaluate effects of using a plethora of pen enrichments (barrier perches, angular ramps, horizontal platforms, large distance between feed and water and providing live Black Soldier fly larvae in a dustbathing area) on tibia characteristics, locomotion, leg health and home pen behaviour of fast and slower-growing broiler chickens. The experiment was set up as a 2 x 2 factorial arrangement with a total of 840 male broiler chickens in a complete randomized design (7 pens per treatment and 30 chickens per pen) with the following treatments: 1) pen enrichment (enriched pen or non-enriched pen); 2) broiler strain (fast-growing Ross 308 or slower-growing Hubbard JA 757). Home pen behaviour and use of enrichment were observed. At approximately 1400 and 2200 g body weight, two chickens per pen were randomly selected and slaughtered, to investigate tibia morphological, biophysical and mechanical characteristics and leg health. Pen enrichment positively affected tibia biophysical characteristics, e.g., osseous volume (Δ = 1.8 cm^3^, *P* = 0.003), total volume (Δ = 1.4 cm^3^, *P* = 0.03) and volume fraction (Δ = 0.02%, *P* = 0.002), in both fast and slower-growing chickens, suggesting that pen enrichment particularly affects ossification and mineralization mechanisms. Accordingly, locomotion and active behaviours were positively influenced by pen enrichment. However, pen enrichment resulted in lower body weight gain in both strains, which might be due to higher activity or lower feed intake as a result of difficulties of crossing the barrier perches. Regarding the strain, slower-growing chickens showed consistently more advanced tibia characteristics and more active behaviour than fast-growing chickens. It can be concluded that pen enrichment may lead to more activity and better bone development in both fast and slower-growing chickens.

## Introduction

In the last decades, genetic selection for growth rate and feed efficiency in broiler chickens resulted in significant phenotypic and genotypic changes [1–4]. Despite the fact that this selection has provided numerous advantages e.g., high amount of meat production in a short rearing duration, less environmental pollution and considerable financial benefits for producers, it has also caused some downsides e.g., suboptimal leg health. Suboptimal leg health appears to be related to an imbalance between high growth rate and immature bones and joints [2, 5–8], which can lead to impaired locomotion [2, 6, 8, 9], pain [8, 10], poor welfare [8, 11–13], higher mortality, lower slaughter revenues and significant financial losses [2, 14–17].

A potential strategy to promote leg health and welfare of modern broiler chickens might be to stimulate activity and locomotion, e.g., by pen enrichment [18–21]. Chickens have been using natural perches, platforms, ramps and elevated resting areas as their natural behaviour throughout their history, from wild ancestors to their modern generations [21–23]. This suggests that these types of enrichments are important to fulfil natural behaviors, but current broiler houses mostly lack any form of enrichment. Several studies assessing behaviour showed that fast-growing broiler chickens spend approximately 80% of their lifespan with passive behaviours (e.g., lying, sitting and resting) [4, 18, 24]. The lack of activity, together with a fast growth rate, may impair bone development, which is one of the reasons for suboptimal leg health and lameness [12, 18, 25–27]. It has been shown that activity can be stimulated through an enriched environment, by i.e., a lower stocking density [21, 28–31], placing platforms and/or ramps [21, 23, 32, 33], barrier perches in between feed and water resources [31, 32], large distance between feed and water [18, 33, 34], different dustbathing materials, such as moss-peat [35], and worms or insects in a dustbathing area [36, 37] resulted in lower prevalence of leg disorders, lower mortality rate and better locomotion, although we did not find this in an earlier comparable study [38]. Increasing physical activity and locomotion may thus result in lower incidence of leg problems by stimulating tibia morphological, biophysical and mechanical properties [18, 33, 39–41].

Another potential strategy to promote leg health and welfare is to reduce growth rate of broiler chickens. Fast growth rate and body weight gain in fast-growing broiler chickens are known to be directly associated with several health and welfare issues. For instance, fast-growing broiler chickens demonstrate more leg and locomotion problems than slower-growing broiler chickens [14, 42, 43]. One of the reasons is that the speed of bone development is unable to keep up with this rapidly increased body weight in fast-growing broiler chickens and they have more porous and less mineralised bones than slower-growing broiler chickens [44, 45]. It has been found that slower-growing broiler chickens spent more time on perches and platforms [46, 47], demonstrated better locomotion [24, 26, 46, 48–50], had less hock and leg problems [46, 51] and lower mortality [52] than fast-growing broiler chickens.

It can be hypothesized that pen enrichment positively affects bone development and locomotion in both fast and slower-growing broiler chickens, but that effects might be larger in the fast-growing broiler chickens, because they generally show worse gait, resulting in less locomotion. However, effects of pen enrichment on locomotion and leg problems in slower-growing broiler chickens are hardly investigated. Therefore, the aim of this study was to investigate effects of a combination of different forms of pen enrichment on tibia characteristics, locomotion, leg health and home pen behaviour of both fast and slower-growing broiler chickens.

## Materials and methods

### Experimental design

The experiment was setup as a 2 x 2 factorial arrangement with two strains of broiler chickens (fast-growing and slower-growing) and two different levels of pen enrichment (enriched and non-enriched). A total of 28 pens (7 pens per treatment, each containing 30 male broiler chickens) within a complete randomized design was used. Male chickens were used, because they show in general more leg health problems than female chickens, due to their higher body weight [26, 46, 50]. Fast-growing broiler chickens were reared till day 38 of age, whereas slower-growing broiler chickens were reared till day 49 of age. The experiment was conducted at the research accommodation of Wageningen Bioveterinary Research (Lelystad, The Netherlands). All procedures in this study were approved by the Central Commission on Animal Experiments (The Hague, The Netherlands; approval number: 2016.D-0138.006).

### Animals, rearing and housing management

**Fig 1:**
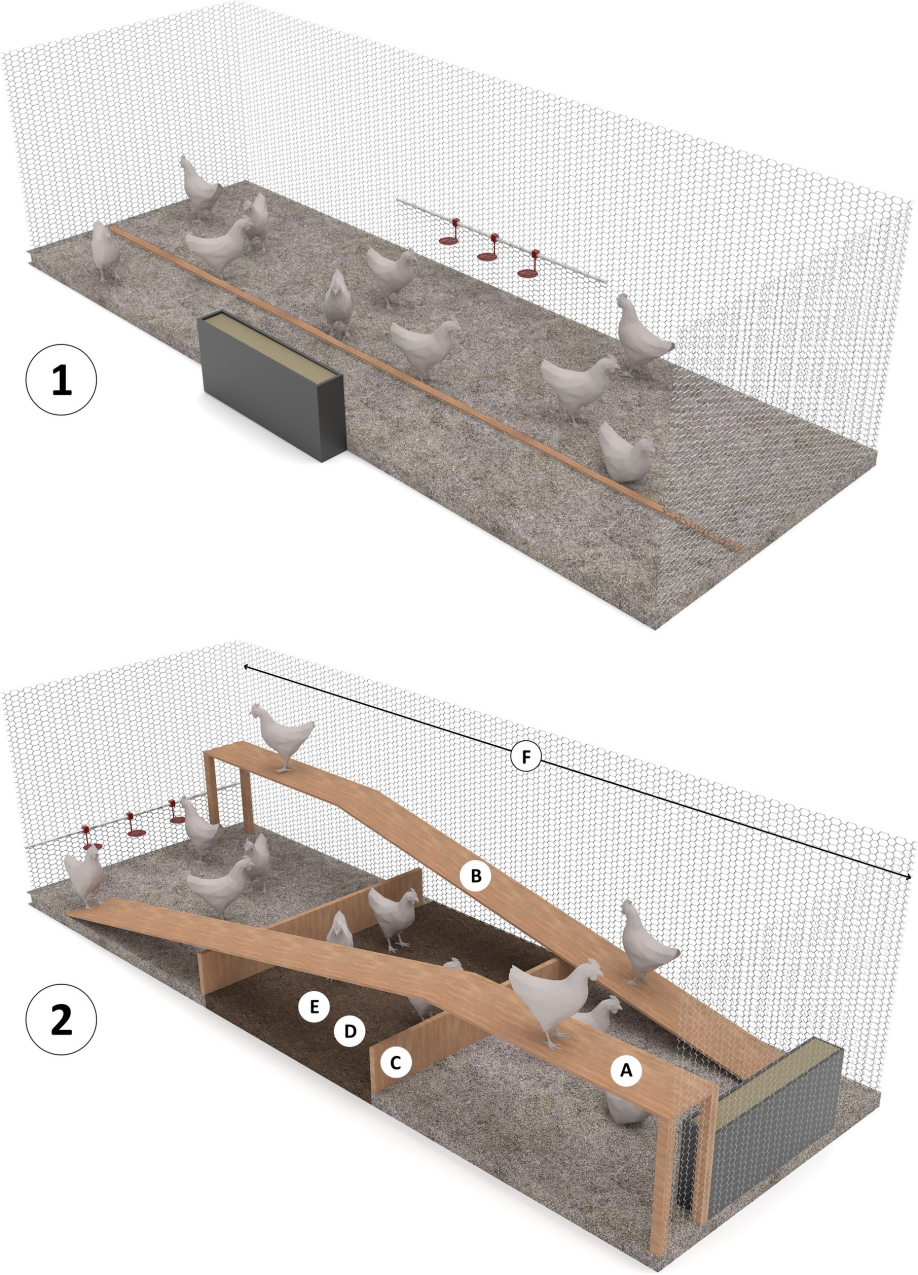
Illustrations of non-enriched (top) and enriched (bottom) pens. Non-enriched pens (3 x 1 m) contained a short distance (1 m) between feeders and drinkers placed on opposite long walls, had one non-adjustable perch and the pen was covered with wood shavings as a bedding material. Enriched pens (3 x 1 m) contained two wooden platforms (A; 100 x 20 x 40 cm, one at each long side of the pen), two wooden ramps (B; 200 x 20 cm, angle of 11.5°), two vertical wooden barrier perches (C; 100 x 4 cm, adjustable in height from 4–16 cm with steps of 4 cm at days 7, 14 and 21), dust bathing area (D; 100 x 100 cm) with peat moss (with depth of 2 cm in week 1, 4 cm in week 2, and 7.5 cm from week 3 onward), provision of live Black Soldier fly larvae (BSFL) in the substrate of the dust bathing area (E; once daily between 11:00 and 11:15 h) and a large distance (3 m) between feeders and drinkers placed on opposite short walls (F). The floor outside the dust bathing area was covered with wood shavings as a bedding material.

A total of 420 fast-growing (Ross 308, breeder age of 30 weeks) and 420 slower-growing (Hubbard JA 757; breeder age of 28 weeks) day-old male broiler chickens were obtained from a commercial hatchery (Probroed, Groenlo, The Netherlands) on the same day and randomly allocated to 28 pens in one broiler house. Half of the chickens per broiler strain were placed in enriched pens, while the other half was placed in non-enriched pens, resulting in the following treatments: enriched fast (**EF**), non-enriched fast (**NF**), enriched slower (**ES**) and non-enriched slower (**NS**). Pen size of both enriched and non-enriched pens was 3 x 1 m and floors in all pens were covered with 4 to 6 cm wood shavings as bedding material. Enriched pens contained two wooden platforms (100 x 20 x 40 cm, one at each long side of the pen), two wooden ramps (200 x 20 cm, angle of 11.5°), a dust bathing area in the centre of the pen (100 x 100 cm) with peat moss (with a thickness of 2 cm in week 1, 4 cm in week 2, and 7.5 cm from week 3 onwards), two vertical wooden barrier perches (100 x 4 cm, adjustable in height from 4 to 16 cm with steps of 4 cm at days 7, 14 and 21), a maximum distance (3 m) between the feeding trough (1 m in length) and 3 nipple drinkers per pen and provision of live Black Soldier fly larvae (**BSFL**) in the substrate of the dust bathing area (once daily between 11:00 and 11:15 h). Because their attractiveness and flavour, all larvae were consumed by chickens in a very short time span (<10 min). The amount of BSFL was determined daily, based on 5% of the expected feed intake, except during the first 7 days, where chickens received a higher level of BSFL (10% on days 0–1, 15% on days 2–4 and 10% on days 5–7). The reason for using higher percentages in these 7 days is related to the number of larvae available for each chicken. With the low feed intake in this phase, only one or two larvae would have been available per chicken in case only 5% BSFL was provided. Non-enriched pens included feed and water (at 1 m distance) and one single long perch (300 x 4 cm, not adjustable in height). The provision of one single perch was done to fulfil the minimal requirements for housing as indicated by the EU directives 2010/63/EU [53] and 2007/526/EC [54]. Illustrations of the enriched and non-enriched pens are provided in Fig 1.

At day 0 (placement), all broilers were provided with a neck tag for individual identification. House temperature was maintained at 34°C at day 0 and gradually decreased to a constant temperature of 18°C at 40 days of age. Relative humidity was kept between 60% and 80% from 1–7 days of age and between 40% and 60% thereafter. The lighting program used was 24L:0D (day 0), 20L:4D (day 1 to 6) and 18L:6D (from day 7 onward, with a continuous dark period during night). At day 0, chickens were vaccinated against infectious bronchitis (eye drop; MSD Animal Health, Boxmeer, The Netherlands) and at day 11, against Newcastle disease (Clone 30; eye drop, MSD Animal Health, Boxmeer, The Netherlands).

Feed and water were provided *ad libitum* for all treatments throughout the whole experiment. Both strains received the same diets with digestible amino acid levels suitable for both strains, based on commercially available diets. A 3-phase feeding program was applied; starter diets were provided from day 0 to 14 (ME_broiler_ = 2925 kcal/kg, CP = 203 g/kg, dLys = 11.1 g/kg), grower diets from day 14 to 35 (ME_broiler_ = 2975 kcal/kg, CP = 171 g/kg, dLys = 9.1 g/kg) and finisher diets from day 35 to 38 (for fast-growing chickens) or 35 to 49 (for slower-growing chickens) (ME_broiler_ = 3025 kcal/kg, CP = 165 g/kg, dLys = 8.6 g/kg). Plant protein based diets were formulated according the energy level and amino acid profile of CVB [55]. Coccidiostats (70 g/kg salinomycin) were added to the grower diet. A protein-fat mixture (consisting of fishmeal and potato protein) (DM = 900 g/kg, ME = 4658 kcal/kg of DM, CP = 419 g/kg of DM, CF = 371 g/kg of DM), with a comparable composition as the BSFL (DM = 350.7 g/kg, ME = 4658 kcal/kg of DM, CP = 419 g/kg of DM, CF = 371 g/kg of DM), was added (mixed with daily feed) to the diet of the non-enriched pens once daily to achieve similar energy and nutrient intake as the broilers in the enriched pens (which received BSFL).

### Data collection, sampling and measurements

All chickens were individually weighed on day 0, 7, 14, 21, 28, 35, 42, and 49 of age. Feed intake (**FI**) was determined per pen at the same days by subtracting the left-overs in the feeder from the amount of feed provided. Body weight (**BW**), FI and feed conversion ratio (**FCR**) were calculated for the three phases and over the whole growing period, taking mortality into account. FI was calculated without BSFL intake of chickens in enriched pens and by excluding the intake of the protein-fat mixture of chickens in non-enriched pens. Mortality was recorded per pen per day. At day 29 and 38, two fast-growing chickens per pen were selected for slaughtering with an average body weight of 1400 and 2200 g, respectively, whereas at day 38 and 49, two slower-growing chickens per pen were selected for slaughtering with the same body weights. Chickens were subjected to electrical stunning without any neck dislocation and decapitation. It was done by a specially designed electrocution device (H2H Euthanizer, Top Equipment B.V., Tiel, The Netherlands). The working principle of the device is passing through the sudden electricity (230 V) into chicken’s body for euthanizing. Then, Varus-Valgus (**VV**) was scored, after fixating the legs at the hip joint to stretch the leg, by determining the angle between the tibia and the metatarsus for both the left (**VV**^**L**^) and right leg (**VV**^**R**^), using a goniometer. Thereafter, the left leg of each chicken was dissected and assessed by a trained poultry veterinarian in post-mortem evaluation on tibia dyschondroplasia (**TD**), bacterial chondronecrosis with osteomyelitis (**BCO**), epiphyseal plate abnormalities (**EPA**), and epiphysiolysis (**EPI**). All these leg disorders were scored in the range of 0 (no abnormalities), 1 (minor abnormality) or 2 (severe abnormality).

**Fig 2:**
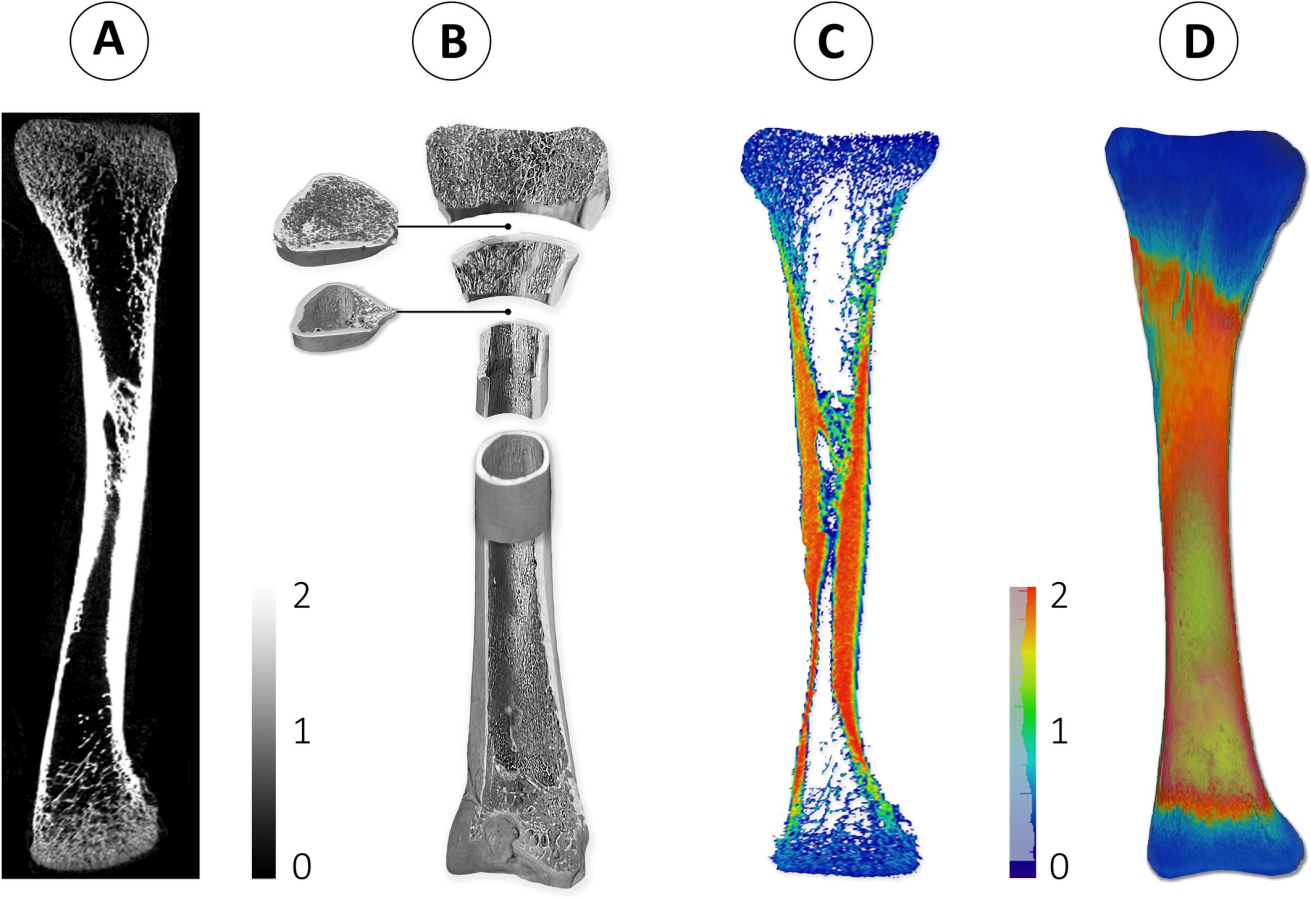
Illustrations of scanned bone by GE phoenix 3D X-ray microfocus CT scanner visualized in avizo 3D viewer software. A) Two-dimensional black and white (grey scale) tibia middle slice view. Shades of grey represent the mineralization areas of bone from dark grey (less mineralization) to white (more mineralization). B) Three-dimensional black and white (grey scale) tibia inner and outer view. C) Three-dimensional coloured tibia outer layer view. Colour scale represents the mineralization areas of outer bone from dark blue (less mineralization, 0) to red (more mineralization). D) Three-dimensional coloured tibia middle slice view. Colour scale represents the mineralization areas of bone from blue (less mineralization, 0) to green (more mineralization, 2).

Right legs were deboned and tibias were packed and frozen at -20°C. After thawing, tibia weight was determined. Tibia proximal length, lateral cortex thickness, femoral and metatarsal side proximal head thickness, osseous volume, pore volume, total volume (osseous volume + pore volume), volume fraction (osseous volume / total volume), mineral content and mineral density were analysed on individual tibia, using a GE Phoenix 3D X-ray microfocus CT scanner (General Electric Company^®^, Boston, Massachusetts, US); for details see [56, 57]. Illustrations of scanned bones are provided in Fig 2. Robusticity index was calculated using the following formula [58]:

Robusticityindex(cm/g)=boneproximallength(cm)/boneweight(g).

The same tibia bones used for 3D X-ray scanning were subjected to a three-point bending test, of which the method is described by [59], using an Instron^®^ electromechanical universal testing machine (Instron^®^, Norwood, Massachusetts, United States). Maximal load of breaking point was used as the tibia ultimate strength; reached yield load just before the angle has changed on slope data was used as the tibia yield strength; the slope of the selected linear part of the curve data was used as the tibia stiffness; the area under the curve of selected region data was used as the tibia energy to fracture. Elastic modulus (GPa), which is the amount of strain as a result of a particular amount of stress [60], was calculated using the following formula [60, 61]:

$$E=\frac{N\;S^{3}}{4\delta \;TL^{3}}$$

where *E* is the elastic modulus (GPa), *N* is the maximal load (N), S is the span between bending fixtures (mm), T is the tibia thickness (mm), L is the tibia length (mm), and δ is the maximum deflection (mm) at the midpoint of the bone.

Gait score of 4 randomly selected chickens per pen was evaluated by an experienced evaluator (blinded to the treatments by carrying the chickens to the observation place and preventing the observer to see the enriched and non-enriched pens) on day 27 (fast-growing chickens) and day 35 (slower-growing chickens), to eliminate BW difference. Gait was scored within a range of 0 (normal locomotion) to 5 (unable to walk) [59]. One more observation day for slower-growing chickens was originally planned on day 48 to make the comparison with the fast-growing chickens. However, due to solid Covid-19 rules at the last week of the experiment, this activity was skipped.

Home pen behaviour (all chickens per pen) and use of enrichment (all chickens per pen in enriched pens) were scored by direct observation of one experienced observer, using instantaneous scan sampling [62] at day 8, 22, 29 and 43. At these days, broilers were observed in their home pen at four moments (8:30, 10:30, 13:00 and 15:00 h). These four scans were considered representative for their behaviour during the day. In enriched pens, half an hour while BSFL feeding was excluded from the observations. To avoid systematic error due to time effect, observation of each round was started from a different pen. On day 43, only slower-growing chickens were present. Per scan per day per pen, behaviour of all chickens was scored during 3 to 4 min. The number of chickens performing the following behaviours was scored: eating, drinking, walking, standing, sitting, comfort behaviour, foraging, dustbathing, ground pecking, aggression and others [38]. Others was defined as chickens demonstrating a behaviour other than all other behaviours described above. After observing the behaviour in a pen, the number of chickens performing the following activities was scored for use of enrichment: chickens on platforms and ramps, chickens under platforms and ramps, dustbathing chickens and chickens perching on barrier.

### Statistical analysis

All statistical analyses were performed in SAS (Version 9.4, 2013, SAS Institute Inc., Cary, North Carolina, US). Model assumptions were approved at both means and residuals for continuous data. Non-normal distributed data were log-transformed before analyses. Pen was used as the experimental unit for all analyses.

All growth performance data from day 0 to 35 (BW, FI, FCR, mortality) were subjected to general mixed model analysis, using the MIXED procedure with model 1.

Y=μ+enrichment+strain+enrichment*strain+Ɛ; (1)

where Y = the dependent variable, μ = the overall mean, enrichment = whether or not pen enrichment was applied (enriched or non-enriched), strain = broiler strain (fast-growing Ross 308 or slower-growing Hubbard JA757), interaction = 2-way interaction between enrichment and strain, Ɛ = residual error.

From day 35 onwards, only chickens from the slower-growing strain were present and growth performance data (BW, FI, FCR, mortality) was subjected to general linear mixed model analysis, using the MIXED procedure with model 2.

Y=μ+enrichment+Ɛ; (2)

where Y = the dependent variable, μ is the overall mean, enrichment = whether or not pen enrichment was applied (enriched or non-enriched), Ɛ = residual error.

Tibia morphological, biophysical and mechanical characteristics, at two body weight classes (1400 and 2200 g), were subjected to general linear mixed model analysis, using the MIXED procedure with model 1.

Home pen behaviour (eating, drinking, walking, standing, sitting, comfort behaviour, foraging, dustbathing, ground pecking, aggression and others) was subjected to general linear mixed model analysis, using the MIXED procedure with model 1 (home pen behaviour at day 8, 22 and 29) and model 2 (home pen behaviour at day 43). Preliminary analyses demonstrated a lack of interaction between strain and enrichment for home pen behaviour and consequently data is presented for only main effects.

Gait score was assessed, using the GLIMMIX procedure with model 1. Gait score at day 27 (fast-growing chickens) and day 35 (slower-growing chickens), when they had similar body weights, were compared.

Use of enrichment (chickens on platforms and ramps, chickens under platforms and ramps, dustbathing chickens and chickens perching on barriers) was subjected to general linear mixed model analysis, using the MIXED procedure with model 3.

Y=μ+strain+Ɛ; (3)

where Y = the dependent variable, μ is the overall mean, strain = broiler breeder strain (fast-growing Ross 308 or slower-growing Hubbard JA757), Ɛ = residual error.

To eliminate BW effect between the fast and slower-growing strain, home pen behaviour and enrichment use at day 22 for fast-growing chickens and day 29 for slower-growing chickens, when they had similar body weights, were compared, using model 1 (home pen behaviour) or model 3 (enrichment).

VV^R^ and VV^L^ were subjected to general linear mixed model analysis, at two body weight classes (1400 and 2200 g), using the MIXED procedure with model 1. Leg disorders (TD, EPA, BCO and EPI) were subjected to generalized linear mixed model analysis, at two body weight classes (1400 and 2200 g), using the GLIMMIX procedure with model 1. Leg disorders were scored as 0 (no abnormalities), 1 (minor abnormality), or 2 (severe abnormality), but analyzed as 0 (no abnormalities) or 1 (abnormalities present). EPA, BCO and EPI were not statistically analysed, because there were only three chickens scored with BCO and no observations at all were recorded for EPA and EPI.

Results are provided as LSmeans ± SEM, unless indicated otherwise. When multiple comparisons were performed, the level of significance was corrected, using Bonferroni. Effects were considered to be significant at P≤0.05.

## Results

### Performance

No interaction effects between enrichment and strain were found on BW (Table 1). Chickens in non-enriched pens had a higher BW than chickens in enriched pens at day 21 (Δ = 35 g, *P* = 0.02), 28 (Δ = 62 g, *P* = 0.007), 35 (Δ = 99 g, *P* = 0.003), 42 (slower-growing chickens only; Δ = 84 g, *P* = 0.003) and 49 (slower-growing chickens only; Δ = 93 g, *P* = 0.005). Slower-growing broilers had a lower BW than fast-growing broilers at day 0 (Δ = 1.8 g), 7 (Δ = 29 g), 14 (Δ = 134 g), 21 (Δ = 321 g), 28 (Δ = 540 g) and 35 (Δ = 822 g) (all *P*<0.001).

**Table 1:** Effects of pen enrichment (enriched or non-enriched), broiler strain (fast-growing ross 308 or slower-growing Hubbard JA 757) and their interaction on body weight (g) of male broiler chickens at different ages (n = 7 pens per treatment, LSmeans±SEM).

Parameter	Day 0	Day 7	Day 14	Day 21	Day 28	Day 35	Day 42^1^	Day 49^1^
Enrichment								
Enriched	36.9	124	334	657^b^	1070^b^	1596^b^	-	-
Non-enriched	36.9	126	346	692^a^	1132^a^	1695^a^	-	-
SEM	0.1	3	6	10	15	20	-	-
Strain								
Fast	37.8^a^	140^a^	407^a^	835^a^	1371^a^	2057^a^	-	-
Slower	36.0^b^	111^b^	273^b^	514^b^	831^b^	1235^b^	-	-
SEM	0.1	3	6	10	15	20	-	-
Enrichment*strain								
Enriched fast	37.8	140	401	817	1324	1985	-	-
Non-enriched fast	37.8	140	413	854	1419	2129	-	-
Enriched slower	36.0	108	267	497	817	1208	1641^b^	2144^b^
Non-enriched slower	36.0	113	279	530	845	1261	1724^a^	2237^a^
SEM	0.2	4	8	14	21	29	15	19
*P*-values								
Enrichment	0.78	0.60	0.17	0.02	0.007	0.003	0.003	0.005
Strain	<0.001	<0.001	<0.001	<0.001	<0.001	<0.001	-	-
Enrichment*strain	0.88	0.55	0.99	0.88	0.13	0.13	-	-

^a-b^Values within a column and factor lacking a common superscript differ (P≤0.05).

^1^At day 42 and 49, only chickens of the slower-growing strain were present.

Neither any interaction effects between pen enrichment and strain nor main pen enrichment effects were found on FI (Table 2). Slower-growing chickens had a lower FI than fast-growing broilers between day 0–14 (Δ = 112 g), day 14–35 (Δ = 923 g) and day 0–35 (Δ = 1034 g) (all *P*<0.001).

**Table 2:** Effects of pen enrichment (enriched or non-enriched), broiler strain (fast-growing ross 308 or slower-growing Hubbard JA 757) and their interaction on feed intake (FI; g per chicken) and feed conversion ratio (FCR = FI/BWG^1^) of male broiler chickens in different phases of the rearing period (n = 7 pens per treatment, LSmeans±SEM).

Parameter	FI	FI	FI	FI	FI	FCR	FCR	FCR	FCR	FCR
	d	d	d	d	d	d	d	d	d	d
	0–14	14–35	0–35	35–49^2^	0–49^2^	0–14	14–35	0–35	35–49^2^	0–49^2^
Enrichment										
Enriched	350	2050	2400	-	-	1.20^a^	1.62^a^	1.54^a^	-	-
Non-enriched	344	2110	2470	-	-	1.13^b^	1.57^b^	1.49^b^	-	-
SEM	7	22	27	-	-	0.01	0.01	0.01	-	-
Strain										
Fast	403^a^	2528^a^	2930^b^	-	-	1.10^b^	1.53^b^	1.45^b^	-	-
Slower	291^b^	1605^b^	1896^a^	-	-	1.23^a^	1.67^a^	1.58^a^	-	-
SEM	7	22	27	-	-	0.01	0.01	0.01	-	-
Enrichment*strain										
Enriched fast	408	2480	2882	-	-	1.13	1.56	1.48	-	-
Non-enriched fast	398	2574	2971	-	-	1.07	1.50	1.42	-	-
Enriched slower	291	1589	1895	2029	3909	1.26	1.69	1.61	2.17	1.85^a^
Non-enriched slower	291	1620	1916	2037	3948	1.20	1.65	1.56	2.09	1.79^b^
SEM	10	31	38	14	23	0.01	0.01	0.01	0.02	0.01
*P*-values										
Enrichment	0.60	0.15	0.29	0.72	0.26	<0.001	<0.001	<0.001	0.06	<0.001
Strain	<0.001	<0.001	<0.001	-	-	<0.001	<0.001	<0.001	-	-
Enrichment*strain	0.58	0.63	0.81	-	-	0.74	0.47	0.53	-	-

^a-b^Values within a column and factor lacking a common superscript differ (P≤0.05).

^1^Body weight gain.

^2^Only slower-growing chickens.

*FI was calculated without BSFL intake of chickens in enriched pens and by excluding the intake of the dietary supplement of chickens in non-enriched pens.

No interaction effects between enrichment and strain were found on FCR (Table 2). Chickens in non-enriched pens had a lower FCR than chickens in enriched pens between days 0–14 (Δ = 0.07), days 14–35 (Δ = 0.05), days 0–35 (Δ = 0.05) and days 0–49 (Δ = 0.05) (all *P*<0.001). Slower-growing chickens had a higher FCR than fast-growing chickens between days 0–14 (Δ = 0.13), 14–35 (Δ = 0.14) and 0–35 (Δ = 0.13) (all *P*<0.001).

A total of 33 (3.9%) dead chickens were recorded during the rearing period. No interaction effects between enrichment and strain or main effects were found on mortality. No chickens were culled.

### Tibia morphological characteristics

At the 1400 g BW class, no interaction effects between pen enrichment and strain were found on tibia morphological characteristics (Table 3) and neither pen enrichment effects were found. Slower-growing chickens had a higher femoral (Δ = 0.17 cm, *P* = 0.02) and metatarsal side proximal tibia head thicknesses (Δ = 0.12 cm, *P* = 0.04) than fast-growing chickens. At the 2200 gBW class, no interaction effects between enrichment and strain were found on tibia morphological characteristics (Table 3) and neither pen enrichment effects were found. Slower-growing broilers had a higher tibia weight (Δ = 0.81 g, *P* = 0.02), proximal tibia length (Δ = 0.63 cm, *P* = 0.008) and metatarsal side proximal tibia head thicknesses (Δ = 0.17 cm, *P* = 0.002) than fast-growing broilers.

**Table 3:** Effects of pen enrichment (enriched or non-enriched), broiler strain (fast-growing ross 308 or slower-growing Hubbard JA 757) and their interaction on tibia morphological characteristics of male broiler chickens in two body weight classes (1400 and 2200 g) (2 chickens per pen, n = 7 pens per treatment, LSmeans±SEM).

Parameter	Tibia weight (g)		Proximal tibia length (cm)		Lateral tibia cortex thickness (cm)		Femoral side proximal tibia head thickness (cm)		Metatarsal side proximal tibia head thickness (cm)		Tibia robusticity index (cm/g)	
BW Class	1400 g	2200 g	1400 g	2200 g	1400 g	2200 g	1400 g	2200 g	1400 g	2200 g	1400 g	2200 g
Enrichment												
Enriched	12.74	14.37	9.54	11.71	1.26	1.37	3.56	3.79	3.20	3.42	0.75	0.82
Non-enriched	12.39	14.04	9.02	11.69	1.22	1.35	3.54	3.81	3.14	3.34	0.73	0.83
SEM	0.32	0.22	0.20	0.15	0.02	0.01	0.05	0.04	0.04	0.03	0.01	0.01
Strain												
Fast	12.27	13.80^b^	9.15	11.38^b^	1.23	1.35	3.46^b^	3.76	3.11^b^	3.30^b^	0.75	0.83
Slower	12.86	14.61^a^	9.41	12.01^a^	1.25	1.37	3.63^a^	3.84	3.23^a^	3.47^a^	0.74	0.82
SEM	0.32	0.22	0.20	0.15	0.02	0.01	0.05	0.04	0.04	0.03	0.01	0.01
Enrichment*strain												
Enriched fast	12.48	13.96	9.38	11.47	1.24	1.36	3.44	3.77	3.13	3.37	0.76	0.82
Non-enriched fast	12.05	13.64	8.92	11.29	1.22	1.34	3.49	3.74	3.09	3.22	0.74	0.83
Enriched slower	12.99	14.78	9.71	11.94	1.27	1.38	3.69	3.81	3.27	3.47	0.75	0.81
Non-enriched slower	12.72	14.44	9.12	12.09	1.23	1.36	3.58	3.87	3.19	3.46	0.72	0.84
SEM	0.45	0.31	0.29	0.22	0.03	0.01	0.07	0.06	0.05	0.05	0.01	0.01
*P*-values												
Enrichment	0.45	0.30	0.09	0.93	0.24	0.15	0.72	0.77	0.30	0.12	0.08	0.18
Strain	0.20	0.02	0.37	0.008	0.47	0.11	0.02	0.19	0.04	0.002	0.35	0.86
Enrichment*strain	0.86	0.99	0.83	0.46	0.75	0.88	0.24	0.45	0.69	0.16	0.58	0.36

^a-b^Values within a column and factor lacking a common superscript differ (P≤0.05).

### Tibia biophysical characteristics

At the 1400 g BW class, no interaction effects between pen enrichment and strain were found on tibia biophysical characteristics (Table 4) and neither pen enrichment effects were found. Slower-growing broilers had a higher tibia osseous volume (Δ = 6.4 cm^3^, *P*<0.001), tibia total volume (Δ = 6.6 cm^3^, *P*<0.001), tibia volume fraction (Δ = 0.04%, *P*<0.001) and tibia mineral content (Δ = 1.1 g, *P*<0.001) than fast-growing broilers. At the 2200 g BW class, an interaction between pen enrichment and strain was found on tibia pore volume (Table 4). Enriched slower-growing group resulted in a lower tibia pore volume compared to other three groups (Δ = 1.0 cm^3^ on average; *P* = 0.02). Chickens in non-enriched pens had a lower tibia osseous volume (Δ = 1.8 cm^3^, *P* = 0.003), tibia total volume (Δ = 1.4 cm^3^, *P* = 0.03) and tibia volume fraction (Δ = 0.02%, *P* = 0.002) than chickens in enriched pens. Slower-growing broilers had a higher tibia osseous volume (Δ = 5.9 cm^3^, *P*<0.001), tibia total volume (Δ = 5.4 cm^3^, *P*<0.001), tibia volume fraction (Δ = 0.05%, *P*<0.001), tibia mineral content (Δ = 0.7 g, *P* = 0.02) and tibia mineral density (Δ = 0.05 g/cm^3^, *P*<0.001) than fast-growing broilers.

**Table 4:** Effects of pen enrichment (enriched or non-enriched), broiler strain (fast-growing ross 308 or slower-growing Hubbard JA 757) and their interaction on tibia biophysical characteristics of male broiler chickens in two body weight classes (1400 and 2200 g) (2 chickens per pen, n = 7 pens per treatment, LSmeans±SEM).

Parameter	Tibia osseous volume (cm^3^)		Tibia pore volume (cm^3^)		Tibia total volume (cm^3^)		Tibia volume fraction (OV^1^/TV^2^)		Tibia mineral content (g)		Tibia mineral density (g/cm^3^)	
BW Class	1400 g	2200 g	1400 g	2200 g	1400 g	2200 g	1400 g	2200 g	1400 g	2200 g	1400 g	2200 g
Enrichment												
Enriched	21.3	25.3^a^	3.5	3.9	24.8	29.2^a^	0.86	0.86^a^	11.2	12.5	0.21	0.37
Non-enriched	20.7	23.5^b^	3.8	4.3	24.5	27.8^b^	0.84	0.84^b^	11.3	12.0	0.19	0.37
SEM	0.6	0.4	0.2	0.1	0.6	0.4	0.01	0.01	0.2	0.2	0.01	0.01
Strain												
Fast	17.8^b^	21.4^a^	3.5	4.4	21.3^b^	25.8^b^	0.83^b^	0.83^b^	10.7^b^	11.9^b^	0.19	0.34^b^
Slower	24.2^a^	27.3^b^	3.7	3.9	27.9^a^	31.2^a^	0.87^a^	0.88^a^	11.8^a^	12.6^a^	0.21	0.39^a^
SEM	0.6	0.4	0.2	0.1	0.6	0.4	0.01	0.01	0.2	0.2	0.01	0.01
Enrichment*strain												
Enriched fast	18.6	22.5	3.5	4.4^a^	22.1	26.9	0.84	0.84	10.5	12.0	0.20	0.35
Non-enriched fast	17.0	20.3	3.6	4.4^a^	20.6	24.7	0.83	0.82	10.9	11.9	0.18	0.34
Enriched slower	24.0	28.0	3.5	3.4^b^	27.4	31.5	0.87	0.89	11.9	12.9	0.22	0.39
Non-enriched slower	24.5	26.6	3.9	4.3^a^	28.4	30.9	0.86	0.86	11.7	12.2	0.21	0.40
SEM	0.8	0.5	0.2	0.2	0.9	0.6	0.01	0.01	0.2	0.2	0.01	0.01
*P*-values												
Enrichment	0.51	0.003	0.18	0.02	0.79	0.03	0.10	0.002	0.65	0.07	0.21	0.78
Strain	<0.001	<0.001	0.50	0.008	<0.001	<0.001	<0.001	<0.001	<0.001	0.02	0.08	<0.001
Enrichment*strain	0.20	0.50	0.51	0.02	0.19	0.16	0.74	0.17	0.31	0.26	0.59	0.19

^a-b^Values within a column and factor lacking a common superscript differ (P≤0.05).

^1^Osseous volume.

^2^Total volume.

### Tibia mechanical characteristics

At the 1400 g BW class, no interaction effects between pen enrichment and strain were found on tibia mechanical characteristics and neither pen enrichment effects were found (Table 5). Slower-growing broilers had a higher tibia ultimate strength (Δ = 21.7 N, *P*<0.001), tibia yield strength (Δ = 21.0 N, *P*<0.001), tibia stiffness (Δ = 20.6 N/mm, *P*<0.001) and tibia energy to fracture (Δ = 21.9 N-mm, *P*<0.001) than fast-growing broilers. At the 2200 g BW class, no interaction effects between pen enrichment and strain were found on tibia mechanical characteristics (Table 5) and neither pen enrichment effects were found. Slower-growing chickens had a higher tibia ultimate strength (Δ = 19.4 N, *P*<0.001), tibia yield strength (Δ = 17.8 N, *P*<0.001), tibia stiffness (Δ = 21.7 N/mm, *P*<0.001) and tibia energy to fracture (Δ = 20.9 N-mm, *P*<0.001) than fast-growing broilers.

**Table 5:** Effects of pen enrichment (enriched or non-enriched), broiler breeder strain (fast-growing ross 308 or slower-growing Hubbard JA 757) and their interaction on tibia mechanical characteristics of male broiler offspring in two body weight classes (1400 and 2200 g) (2 chickens per pen, n = 7 pens per treatment, LSmeans±SEM).

Parameter	Tibia ultimate strength (N)		Tibia yield strength (N)		Tibia stiffness (N/mm)		Tibia energy to fracture (N-mm)		Tibia elastic modulus (GPa)	
BW Class	1400 g	2200 g	1400 g	2200 g	1400 g	2200 g	1400 g	2200 g	1400 g	2200 g
Enrichment										
Enriched	242.4	274.6	226.4	256.1	233.5	269.1	231.4	261.9	12.6	12.2
Non-enriched	237.2	271.2	223.4	254.3	228.6	263.8	226.8	260.2	12.4	12.0
SEM	2.2	1.8	2.1	2.1	2.1	1.9	2.0	1.9	0.4	0.3
Strain										
Fast	229.0^b^	263.2^b^	214.4^b^	246.3^b^	220.7^b^	255.6^b^	218.1^b^	250.6^b^	12.4	12.6
Slower	250.6^a^	282.6^a^	235.4^a^	264.1^a^	241.3^a^	277.3^a^	240.0^a^	271.5^a^	12.6	11.6
SEM	2.2	1.8	2.1	2.1	2.1	1.9	2.0	1.9	0.4	0.3
Enrichment*strain										
Enriched fast	229.0	263.4	213.5	246.2	220.6	258.9	217.9	250.5	12.4	13.0
Non-enriched fast	229.1	263.0	215.4	246.4	220.9	252.3	218.3	250.7	12.4	12.1
Enriched slower	255.9	285.7	239.3	266.0	246.4	279.3	244.8	273.3	12.7	11.5
Non-enriched slower	245.4	279.4	231.5	262.1	236.3	275.3	235.3	269.8	12.5	11.8
SEM	3.1	2.6	2.9	3.0	2.9	2.7	2.9	2.7	0.6	0.4
*P*-values										
Enrichment	0.11	0.22	0.32	0.55	0.11	0.06	0.13	0.55	0.83	0.55
Strain	<0.001	<0.001	<0.001	<0.001	<0.001	<0.001	<0.001	<0.001	0.78	0.06
Enrichment*strain	0.10	0.27	0.11	0.49	0.09	0.64	0.10	0.49	0.90	0.18

^a-b^Values within a column and factor lacking a common superscript differ (P≤0.05).

### Leg disorders and gait score

No interaction effects between pen enrichment and broiler breeder strain were found on VV^R^, VV^L^ and TD at both 1400 and 2200 g BW classes and no main effects of pen enrichment or strain were found on TD. At the 1400 g BW class, VV angulation was not affected by enrichment or strain (on average 4.90^o^). At the 2200 g BW class, slower-growing chickens had a lower VV^R^ than fast-growing chickens (3.80° vs 6.04°, respectively, *P* = 0.003). VV^L^ angulation was not affected by enrichment or strain at this BW class (on average 5.88^o^). No interaction and main effects were found on TD, which was observed in 11 chickens (9.82%) at 1400 g BW class and 10 chickens (8.92%) at 2200 BW class.

At similar BW class (day 27 for fast-growing chickens and day 35 for slower-growing chickens), no interaction between pen enrichment and strain (*P* = 0.97) and neither main effects of pen enrichment (*P* = 0.97) or strain (*P* = 1.00) were found on gait score.

### Home pen behaviour and use of enrichment

Chickens in non-enriched pens showed less foraging behaviour at day 8 (Δ = 8.47%, *P*<0.001), day 22 (Δ = 9.19%, *P*<0.001), day 29 (Δ = 5.6%, *P*<0.001) and day 43 (slower-growing chickens only, Δ = 8.86%, *P*<0.001), less dust bathing behaviour at day 8 (Δ = 0.97%, *P* = 0.006) and less ground pecking behaviour at day 43 (slower-growing chickens only, Δ = 3.98%, *P* = 0.02) than chickens in enriched pens (Table 6). The opposite was found for standing behaviour at day 29 (Δ = 4.15%, *P* = 0.007), sitting behaviour at day 22 (Δ = 8.97%, *P* = 0.03), ground pecking behaviour at day 8 (Δ = 3.71%, *P* = 0.002) and aggression behaviour at day 29 (Δ = 1.05%, *P* = 0.02), which behaviours were all higher in non-enriched pens than in enriched pens. Slower-growing chickens showed more walking behaviour at day 8 (Δ = 6.99%, *P* = 0.001), day 22 (Δ = 7.6%, *P*<0.001) and day 29 (Δ = 5.32%, *P*<0.001), more standing behaviour at day 8 (Δ = 2.34%, *P* = 0.03), day 22 (Δ = 3.12%, *P* = 0.02) and day 29 (Δ = 8.91%, *P*<0.001), more foraging behaviour at day 22 (Δ = 6.61%, *P* = 0.009) and day 29 (Δ = 3.9%, *P* = 0.002) and more aggression behaviour at day 22 (Δ = 0.94%, *P* = 0.03) and day 29 (Δ = 1.02%, *P* = 0.03) than fast-growing chickens. The opposite was found for eating behaviour at day 8 (Δ = 3.04%, *P* = 0.04) and day 22 (Δ = 1.16%, *P* = 0.03) and sitting behaviour at day 22 (Δ = 19.28%, *P*<0.001) and day 29 (Δ = 8.91%, *P*<0.001).

**Table 6:** Effects of pen enrichment (enriched or non-enriched) and broiler strain (fast-growing ross 308 or slower-growing Hubbard JA 757) on percentage of male chickens showing eating, drinking, walking, standing, sitting, comfort behaviour, foraging, dustbathing, ground pecking, aggression and other behaviours at day 8, 22, 29 and 43 of age (n = 7 pens per treatment; LSmeans±SEM).

Parameter and day (%)	Pen enrichment		Strain		SEM	*P*-values^2^	
	Enriched	Non-enriched	Fast	Slower		Enrichment	Strain
Eating							
Day 8	6.90	6.06	8.00^a^	4.96^b^	0.94	0.54	0.04
Day 22	1.65	1.80	2.31^a^	1.15^b^	0.33	0.75	0.03
Day 29	1.78	2.78	2.86	1.69	0.46	0.14	0.09
Day 43^1^	1.65	1.79	-	-	0.51	0.86	-
Drinking							
Day 8	13.17	12.01	13.76	11.42	1.58	0.61	0.31
Day 22	5.33	5.58	5.67	5.24	0.72	0.81	0.69
Day 29	4.36	4.78	5.00	4.13	0.72	0.69	0.40
Day 43^1^	3.78	3.23	-	-	0.58	0.51	-
Walking							
Day 8	13.42	12.27	9.35^b^	16.34^a^	1.10	0.47	0.001
Day 22	7.92	7.09	3.70^b^	11.30^a^	1.22	0.64	<0.001
Day 29	5.67	6.90	3.62^b^	8.94^a^	0.82	0.31	<0.001
Day 43^1^	7.99	8.56	-	-	1.46	0.80	-
Standing							
Day 8	5.68	7.65	5.50^b^	7.84^a^	0.72	0.07	0.03
Day 22	4.54	5.44	3.43^b^	6.55^a^	0.81	0.44	0.02
Day 29	5.50^b^	9.65^a^	3.12^b^	12.03^a^	1.00	0.007	<0.001
Day 43^1^	13.64	15.84	-	-	1.46	0.29	-
Sitting							
Day 8	32.57	37.36	37.61	33.31	2.44	0.18	0.14
Day 22	45.61^b^	54.58^a^	59.73^a^	40.45^b^	2.72	0.03	<0.001
Day 29	57.36	55.23	64.78^a^	47.80^b^	2.90	0.61	<0.001
Day 43^1^	50.06	58.22	-	-	3.97	0.18	-
Comfort behaviour							
Day 8	7.55	9.34	7.15	9.74	1.00	0.22	0.08
Day 22	8.86	10.12	8.84	10.13	1.08	0.42	0.41
Day 29	6.19	7.60	7.27	6.52	1.12	0.39	0.64
Day 43^1^	4.46	3.65	-	-	0.95	0.56	-
Foraging							
Day 8	14.45^a^	5.98^b^	9.67	10.76	1.00	<0.001	0.45
Day 22	12.88^a^	3.69^b^	4.98^b^	11.59^a^	1.64	<0.001	0.009
Day 29	8.50^a^	2.90^b^	3.75^b^	7.65^a^	0.79	<0.001	0.002
Day 43^1^	9.78^a^	0.92^b^	-	-	1.25	<0.001	-
Dust bathing							
Day 8	1.09^a^	0.12^b^	0.90	0.30	0.23	0.006	0.07
Day 22	0.65	0.44	0.65	0.44	0.24	0.56	0.54
Day 29	0.46	0.67	0.36	0.77	0.28	0.60	0.31
Day 43^1^	0.28	0.15	-	-	0.17	0.59	-
Ground pecking							
Day 8	4.83^b^	8.54^a^	7.40	5.97	0.73	0.002	0.18
Day 22	12.02	10.76	10.60	12.17	1.17	0.46	0.36
Day 29	10.08	8.33	9.09	9.31	1.17	0.31	0.90
Day 43^1^	8.10^a^	4.12^b^	-	-	0.95	0.02	-
Aggression							
Day 8	0.35	0.22	0.22	0.35	0.17	0.60	0.60
Day 22	0.61	0.59	0.14^b^	1.06^a^	0.29	0.97	0.04
Day 29	0.12^b^	1.17^a^	0.14^b^	1.16^a^	0.29	0.02	0.03
Day 43^1^	0.26^b^	3.53^a^	-	-	0.98	0.04	-
Others^3^							
Day 8	-	0.13	0.13	-	0.08	0.26	0.26
Day 22	-	-	-	-	-	-	-
Day 29	-	-	-	-	-	-	-
Day 43^1^	-	-	-	-	-	-	-

^a-b^Values within a factor and row lacking a common superscript differ (*P≤*0.05).

^1^Only slower-growing chickens.

^2^No interactions between broiler breeder strain and pen enrichment were observed for any of the behaviours at any of the sampling days.

^3^Chickens demonstrating a behaviour other than eating, drinking, walking, standing, sitting, comfort behaviour, foraging, dustbathing, ground pecking and aggression.

In enriched pens, a clear strain effect was found on use of different enrichment objects (Table 7). The percentage of chickens on platforms and ramps at day 29 (Δ = 14.6%, *P*<0.001) and perching on barriers at day 8 (Δ = 4.9%, *P*<0.001), day 22 (Δ = 14.05%, *P*<0.001) and day 29 (Δ = 16.05%, *P*<0.001) was higher in slower-growing chickens than in fast-growing chickens. The opposite was found for the percentage chickens under platforms and ramps at day 29 (Δ = 13.72%, *P* = 0.003) and dustbathing chickens at day 8 (Δ = 1.19%, *P* = 0.05).

**Table 7:** Effects of broiler strain (fast-growing ross 308 or slower-growing Hubbard JA 757) on percentage of male chickens in enriched pens, using the following enrichment objects (on platforms and ramps, under platforms and ramps, dustbathing, perching on barriers) at day 8, 22, 29 and 43 of age (n = 7 pens per treatment, LSmeans±SEM).

Parameter and day^1^	Strain		SEM	*P*-values
	Fast	Slower		
Chickens on platforms and ramps				
Day 8	13.69	16.18	2.49	0.50
Day 22	17.98	24.04	2.06	0.06
Day 29	14.69^b^	29.25^a^	1.75	<0.001
Day 43^2^	-	29.75	-	-
Chickens under platforms and ramps				
Day 8	16.95	30.35	4.51	0.06
Day 22	22.74	16.97	2.60	0.15
Day 29	31.48^a^	17.76^b^	1.91	<0.001
Day 43^2^	-	19.55	-	-
Dustbathing chickens				
Day 8	1.56^a^	0.37^b^	0.37	0.05
Day 22	0.4	0.63	0.26	0.54
Day 29	0.14	0.37	0.21	0.47
Day 43^2^	-	0.14	-	-
Chickens perching on barriers				
Day 8	2.08^b^	6.98^a^	0.75	<0.001
Day 22	3.92^b^	17.97^a^	1.53	<0.001
Day 29	3.97^b^	20.02^a^	0.90	<0.001
Day 43^2^	-	17.57	-	-

^a-b^Values within a row lacking a common superscript differ (*P*≤0.05).

^1^The percentages of chickens demonstrating no use of enrichment are not demonstrated in the table.

^2^Only slower-growing chickens.

At similar BW, slower-growing chickens showed more walking (Δ = 5.24%), standing (Δ = 8.6%), foraging (Δ = 2.67%), dust bathing (Δ = 0.12%) and aggression (Δ = 1.02%) behaviour than fast-growing chickens, while the opposite was found for eating (Δ = 0.62%), drinking (Δ = 1.54%), sitting (Δ = 11.93%) and comfort (Δ = 2.32%) behaviour. At similar BW, more slower-growing chickens were on platforms and ramps (Δ = 11.27%) and perching on barriers (Δ = 16.1%) than fast-growing chickens, whereas the opposite was found for percentage of chickens under platforms and ramps (Δ = 4.98%) and dustbathing chickens (Δ = 0.03%).

## Discussion

The aim of this study was to investigate effects of a combination of different forms of pen enrichment on tibia characteristics, locomotion, leg health and home pen behaviour of both fast and slower-growing broiler chickens. Hardly any interactions were found between strain and enrichment, indicating that both fast and slower-growing chickens are both able to use several forms of enrichment in a comparable way.

### Growth performance

Results of the current study showed that pen enrichment resulted in lower body weight and higher FCR in both fast and slower-growing chickens. These findings are supported by recent studies [63, 64], who observed a negative effect of pen enrichment on growth performance. In an earlier comparable study [38], pen enrichment resulted in lower body weight, but also in a higher FI and a lack of effect on FCR. In the latter study, the FI of the non-enriched pens included the protein-fat mixture, where this was excluded in the current study. Results of current study are not in accordance with other studies [34, 65–68], who found no significant effects of pen enrichment on any growth performance parameters. This discrepancy among studies might be related to the fact that less complex pen enrichment forms were used in these studies compared to the current study. For example, only barrier perches [34, 65, 66] and mirror, ball, perch and dust (each material in another pen) [67] were used in these studies. The lower body weight gain of enriched-housed broilers in the current experiment might be related to 1) the comprehensive enrichment design of the current study, which contains a combination of platforms, angular ramps, barrier perches, large distance between feed and water and live Black Soldier fly larvae in the moss-peat dust bathing area. A plethora of different enrichments might exponentially stimulate physical activity and consequently a higher metabolic energy use, which will result in a lower body weight gain. This higher activity in enriched pens is supported by the higher percentage of chickens showing active behaviours and use of enrichment in both fast and slower-growing chickens. 2) Chickens might have had difficulties to cross the barrier perches to access the feed on one side and water on the other side [38]. This might be due to other chickens perching and consequently blocking the way from one side of the pen to the other side. It might also be related to their heavy body weight, particularly in the last week of the rearing period. Whether or not a more balanced pen enrichment might have comparable stimulatory effects on activity, while maintaining performance, needs to be investigated.

Regarding the strain, in the current study, body weight and feed intake of fast-growing chickens were higher than slower-growing broiler chickens on same ages, which is in accordance with previous studies [45, 68–70]. Due to a use of very young broiler breeders, body weight gain and BW at slaughter was relatively low [71, 72], which might have resulted in a low prevalence of leg disorders as well (see below).

### Tibia characteristics

One of the most important underlying reasons for suboptimal bone development in broiler chickens is high growth rate, while low activity levels is the other one [12, 24, 41]. The hypothesis of this study was that leg health and bone characteristics in broiler chickens can be improved through pen enrichment, which has previously been confirmed by several studies [21, 30, 31, 33, 35]. Focusing on bone properties, a higher activity has been found to positively affect tibia morphological, biophysical and mechanical characteristics of chickens [26, 33, 35, 44, 73–75]. A large distance between feed and water resulted in increased walking activity [18] and better tibia development in broiler chickens [26, 33]. Barrier perches resulted in improved tibia characteristics of laying hens [73]. Using sand as a dustbathing material and addition of strings for activity stimulation resulted in better bone development in fast-growing broiler chickens [74].

The results of the current study showed that tibia osseous volume, total volume and volume fraction of both fast and slower-growing broiler chickens and tibia pore volume of slower-growing chickens only were positively affected by pen enrichment, while the other tibia characteristics did not differ between enrichment treatments. These findings are in agreement with previous studies, indicating the stimulating effects of pen enrichment on bone characteristics [33, 75, 76]. Tibia characteristics were found correlated with leg disorders and locomotion. Chickens with advanced tibia characteristics showed better locomotion and less leg disorders [75, 76]. In the current study, it can be suggested that bone mineral deposition is the most stimulated physiological mechanism by pen enrichment, whereas tibia morphological and mechanical characteristics, such as tibia weight, length, strength and stiffness, were not affected. It can be hypothesized that stimulated activity due to pen enrichment particularly affects physiological pathways involved in ossification and mineralization, rather than affecting anatomical and physical tibia characteristics.

Regarding the strain, almost all tibia morphological, biophysical and mechanical characteristics in both body weight classes were higher in slower-growing chickens than in fast-growing chickens. These findings are in line with previous studies, indicating that slower-growing chickens demonstrate better bone characteristics in all ages compared to fast-growing chickens [14, 42, 43, 77, 78]. Fast-growing chickens have more porous and less mineralized leg bones than slower-growing broiler chickens, which together with a higher body weight results in a higher risk of lameness [14, 42–45, 79], impaired activity and locomotion [24, 26, 46, 48, 49, 80] and more leg problems [46, 51].

### Leg disorders and gait score

In the current study, the incidence of TD did not differ between pen enrichment, nor between strains, while other leg disorders (BCO, EPA and EPI) were hardly or not observed. These results might be explained by a relatively low stocking density (10 chickens/m^2^), which is related to a low prevalence of leg disorders [10, 40, 79, 81]. The low stocking density was used according to the EU legislation 2010/63/EU [53]. Additionally, BW of the chickens was relatively low, probably related to the use of offspring from young broiler breeders. VV angulation in right legs was found to be higher in fast-growing chickens than in slower-growing chickens. These results are in line with previous studies, indicating that growth rate plays an important role on the prevalence of VV [12, 82–84]. This might be explained by irregular and poor vascular morphology of the epiphyseal growth plate and insufficiently mineralized bones in fast-growing broiler chickens [83, 85]. Slower-growing chickens, on the contrary, have more time for bone mineralization, which compensates the lack of mineralization in the early growth phase, that loads less stress on the skeleton [72, 82, 86, 87], and eventually result in a low incidence of VV. Despite the fact that VV angulation in right legs differed between strains in the current study, the maximal average angulation was 6.04^o^ and it can be disputed whether or not this degree of angulation can be considered as VV or as a leg disorder. Stimulating locomotion by pen enrichment might be more beneficial for fast-growing chickens, since they have less advanced bone development and poorer leg health than slower-growing chickens [38, 42, 73, 74, 87, 88]. However, in the current study no differences between treatments were found on gait score.

### Home pen behaviour and use of enrichment

Results of home pen behaviour showed that broiler chickens in enriched pens demonstrated higher or a tendency to higher percentages of active behaviours (e.g., standing, walking, foraging) and lower percentages of passive behaviours (e.g., comfort behaviour, sitting) than chickens in non-enriched pens. This is in accordance with previous studies, indicating that pen enrichment may stimulate physical activity. Placing horizontal platforms [21, 23, 33], angular ramps [89] and barrier perches [30–32, 90] resulted in stimulated activity in broiler chickens. Using wooden boxes with peat for dust bathing, two platforms with ramps and two bales of peat as a pen enrichment resulted in more wing flapping, wing stretching, body shaking, ground scratching, ground pecking and foraging behaviours in fast-growing broiler chickens compared to non-enriched pens [91]. Scattering mealworms [36] and Black Soldier fly larvae [37, 38] on the litter in fast-growing broiler chickens resulted in increased physical activity and locomotion. A large distance between feeder and drinker as a pen enrichment also resulted in a high percentage of active behaviours [38, 92, 93]. Different dustbathing materials, such as moss-peat have also been found to contribute to activity of broiler chickens [35, 38].

In the current study, slower-growing broiler chickens demonstrated higher or tendency to higher percentages of active behaviours (e.g., standing, walking, foraging) and lower percentages of passive behaviours (e.g., comfort behaviour, sitting) at all observation days and also on similar body weights (day 22 for fast-growing chickens and day 29 for slower-growing chickens) than fast-growing chickens. In addition, use of enrichment objects differed between fast and slower-growing broiler chickens. The most attention-grabbing difference was observed in chickens perching on barriers. A considerably higher percentage of slower-growing chickens were found perching on barriers compared to fast-growing chickens at all ages and also at the same body weight. Slower-growing chickens also showed a higher or a tendency to higher preference to go on ramps and platforms, while fast-growing chickens preferred to stay under the platforms and ramps. These findings are in in line with previous studies showing slower-growing chickens demonstrated more active behaviours than fast-growing chickens. Fast-growing broiler chickens showed higher percentages of time sitting idle and lower percentages of time standing and walking than slower-growing chickens [26, 45, 47, 49]. Slower-growing chickens have been found to use perches more than fast-growing chickens [30, 38, 94–96]. It has been shown that fast-growing broiler chickens showed a preference for lying and sitting on the litter instead of using raised platforms and perches. All these findings might be due to the imbalance between high growth rate and immature bones of fast-growing chickens than slower-growing chickens, which negatively affects standing, particularly at higher body weights, walking and foraging behaviours. This makes fast-growing chickens have more difficulties with barrier perches to access feed and water, to climb and go down on angular ramps than the slower-growing chickens. Another potential reason for these differences between strains might be related to body weight and heavy breast muscles. However, the current study clearly demonstrates that at the same BW class, still differences in activity related behaviours were present between the fast and slower-growing chickens, which suggests that other aspects than BW appears to play a role as well.

## Conclusion

In both slower and fast-growing chickens, tibia biophysical characteristics were positively influenced by comprehensive pen enrichment, while tibia morphological and mechanical characteristics were not affected, suggesting that pen enrichment particularly affects physiological mechanisms related to ossification and mineralization. Slower-growing chickens showed better tibia characteristics, active behaviours and higher tendencies to use enrichment objects than fast-growing chickens. Pen enrichment resulted in lower body weight gain in both fast and slower-growing chickens. The relationship between tibia development and leg health remains unclear, because of the very low incidence of leg disorders in the current study.
